# Personalized Lens Correction Improves Quantitative Fundus Autofluorescence Analysis

**DOI:** 10.1167/iovs.65.3.13

**Published:** 2024-03-11

**Authors:** Leon von der Emde, Geena C. Rennen, Marc Vaisband, Jan Hasenauer, Raffael Liegl, Monika Fleckenstein, Maximilian Pfau, Frank G. Holz, Thomas Ach

**Affiliations:** 1 Department of Ophthalmology, University Hospital Bonn, Bonn, Germany; 2 University of Bonn, Life and Medical Sciences Institute, Bonn, Germany; 3 Department of Internal Medicine III with Haematology, Medical Oncology, Haemostaseology, Infectiology and Rheumatology, Oncologic Center; Salzburg Cancer Research Institute-Laboratory for Immunological and Molecular Cancer Research (SCRI-LIMCR); Paracelsus Medical University, Salzburg, Austria, Cancer Cluster Salzburg, Austria; 4 Helmholtz Center Munich-German Research Center for Environmental Health, Institute of Computational Biology, Neuherberg, Germany; 5 Department of Ophthalmology and Visual Science, John A. Moran Eye Center, University of Utah, Salt Lake City, Utah, United States; 6 Institute of Molecular and Clinical Ophthalmology Basel, Mittlere Strasse 91, Basel, Switzerland

**Keywords:** quantitative fundus autofluorescence (QAF), multimodal imaging of the lens, Scheimpflug imaging, anterior chamber optical coherence tomography (AC-OCT), quantitative autofluorescence lens

## Abstract

**Purpose:**

Quantitative fundus autofluorescence (QAF) currently deploys an age-based score to correct for lens opacification. However, in elderly people, lens opacification varies strongly between individuals of similar age, and innate lens autofluorescence is not included in the current correction formula. Our goal was to develop and compare an individualized formula.

**Methods:**

One hundred thirty participants were examined cross-sectionally, and a subset of 30 participants received additional multimodal imaging 2-week post-cataract-surgery. Imaging included the Scheimpflug principle, anterior chamber optical coherence tomography (AC-OCT), lens quantitative autofluorescence (LQAF), and retinal QAF imaging. Among the subset, least absolute shrinkage and selection operator regression and backward selection was implemented to determine which lens score best predicts the QAF value after lens extraction. Subsequently, a spline mixed model was applied to the whole cohort to quantify the influence of LQAF and Scheimpflug on QAF.

**Results:**

Age and LQAF measurements were found to be the most relevant variables, whereas AC-OCT measurements and Scheimpflug were eliminated by backward selection. Both an increase in Scheimpflug and LQAF values were associated with a decrease in QAF. The prediction error of the spline model (mean absolute error [MAE] ± standard deviation) of 32.2 ± 23.4 (QAF a.u.) was markedly lower compared to the current age-based formula MAE of 96.1 ± 93.5. Both smooth terms, LQAF (*P* < 0.01) and Scheimpflug (*P* < 0.001), were significant for the spline mixed model.

**Conclusions:**

LQAF imaging proved to be the most predictive for the impact of the natural lens on QAF imaging. The application of lens scores in the clinic could improve the accuracy of QAF imaging interpretation and might allow including aged patients in future QAF studies.

Clinical autofluorescence imaging provides a noninvasive means to visualize the metabolic changes in the retina, especially in the retinal pigment epithelium (RPE). This technique has become indispensable for diagnosing and monitoring a variety of retinal diseases. Building upon this, quantitative fundus autofluorescence (QAF) is an innovative technique that facilitates the measurement and accurate quantification of the retina's autofluorescence intensity.^1^^,^^2^ The unique aspect of QAF is its internal reference, which empowers researchers and clinicians to compare autofluorescence intensities across individuals, regardless of where the data was recorded and the instrument used.^3^ This feature is particularly valuable when examining retinal conditions that impact the RPE and its autofluorescence properties.^4^^,^^5^ For instance, QAF has proven instrumental in studies related to age-related macular degeneration (AMD), where diminished autofluorescence is frequently observed.^6^^,^^7^

QAF imaging, however, heavily depends on the clarity of the optical media, such as the cornea, the lens, and the vitreous.^1^ With increasing age, glycation products accumulate in the lens fiber cells, contributing to its opacity.^2^ This leads to a decrease in the transmission of external light to the retina. Short wavelength blue light (488 nm), as used in QAF imaging, is particularly affected.^3^ This is described as Rayleigh scattering and describes a phenomenon that the intensity of scattered light is inversely proportional to the fourth power of the wavelength (blue/violet shortest wavelength in the visual spectrum). To quantify the impact of the lens on QAF, Greenberg et al. introduced an age-related lens correction factor.^4^ However, previous studies have shown that there are large individual variations in lens opacity within an age cohort.^5^^,^^6^ Therefore, some studies only included phakic participants under the age of 65 years, as the percentage of more pronounced cataract increases in the older population.^4^^,^^7^^,^^8^ This, however, is a major limitation, as many ophthalmological diseases, such as AMD, primarily affect older age groups.^9^

There are different approaches to quantify lens opacity. One possibility is to determine the cataract grade clinically by slit lamp examination using the Lens Opacities Classification System (LOCS) grading score.^10^ The cataract is classified in terms of both its severity and anatomical position, which, however, requires clinical experience of the grader. There are also several lens imaging modalities that are more independent of the examiner. Scheimpflug photography, in conjunction with densitometric image analysis, is able to measure the amount of light that is back-scattered from the lens.^11^ Another possibility of objective lens measurement is swept source anterior chamber optical coherence tomography (AC-OCT) imaging, which measures the reflectivity of the lens.^12^ It is also possible to analyze the intensity of the IVth Purkinje image across different wavelengths to accurately quantify lens density and spectral transmittance.^13^ Further, it is possible to use fluorophotometry measurements deploying blue and green autofluorescence images to measure lens transmission.^14^ This is done by comparing autofluorescence measures of the anterior and posterior parts of the lens and equating the difference in fluorescence between both to be attributed to a loss of exciting and fluorescent light in the lens. Likewise, Charng and colleagues recently described a novel method to measure lens autofluorescence (LQAF) by shifting the focus of the QAF acquisition to the lens.^5^ In this study, we focused on the investigation of mainly three novel lens scores, including Scheimpflug, AC-OCT, and LQAF.

This study aims to characterize the utility of novel lens scores for a personalized lens correction in subjects with the natural lens. Predictions of QAF values will be made using spline mixed models, considering both age and lens scores as impact factors. For validation, QAF values of the retina are measured in patients with phakic lens before and after cataract surgery to compare the age-based and lens score-based approaches. More accurate correction of the impact of the lens on QAF imaging could improve the applicability of QAF for interventional studies.

## Methods

### Participants

One hundred thirty participants without retinal pathologies (age range from 20 to 80 years) were recruited from the Department of Ophthalmology, University Hospital Bonn, between November 2021 and November 2022. The participants received a detailed explanation of the study before giving their written informed consent. The study was approved by the Ethics Committee of the University of Bonn (#385/20), and all study procedures adhered to the Tenets of the Declaration of Helsinki. Inclusion criteria were age ≥18 years without prior cataract surgery. Exclusion criteria were refractive errors ≥5.00 diopters of spherical equivalent and/or >1.50 diopters of astigmatism; any previous intraocular surgery; presence of retinal disease; glaucoma; or relevant anterior segment diseases that lead to media opacities.

### Imaging Protocol

All participants were examined cross-sectionally. A subset of 30 participants, who underwent routine cataract surgery, were re-examined 2 weeks after surgery. Participants were examined clinically, including best corrected visual acuity (BCVA). After the pupil of the study eye was dilatated using 1% tropicamide and 2.5% phenylephrine, a slit lamp examination and multimodal imaging were performed.

The multimodal imaging protocol included Scheimpflug imaging (Oculus Pentacam, Wetzlar, Germany) of the anterior segment in 25 single-slit images using a blue light diode in rotation from 0 degrees to 180 degrees around the eye.^15^ Furthermore, swept-source anterior chamber OCT (AC-OCT, ANTERION Cataract App; Anterion, Heidelberg Engineering, Heidelberg, Germany) was performed using the cataract mode which includes images of the anterior segment of the eye, particularly the cornea, anterior chamber, and lens based on 16.640 A-Scans over the central 8 mm. In addition, a quantitative autofluorescence image of the lens (LQAF) was taken following the study protocol by Charng and colleagues^5^: the focus was set to +45 diopters, and 64 images were obtained over 8 mm using the QAF mode (488-nm excitation, laser power = 100%, sensitivity = 67%, and 30 degrees lens). Other imaging modalities used were combined confocal scanning laser ophthalmoscopy (cSLO) imaging and spectral-domain optical coherence tomography of the macula (SD-OCT; 30 degrees × 25 degrees, ART 25, 121 B-scans, Spectralis HRA-OCT 2; Heidelberg Engineering, Heidelberg, Germany). Last, a QAF image of the fundus was acquired. For that purpose, a series of 12 single frames was taken after bleaching of the photopigment.^4^ The image frames can then be used to create an average QAF image. The internal reference is simultaneously excited and captured during QAF imaging (excitation = 488 nm, emission = 500–750 nm, image size = 30 degrees × 30 degrees, 768 × 768 pixels).^16^ This ensures that laser power and camera settings (i.e. sensitivity), which might differ from examination to examination or subject to subject, can then be normalized to the internal reference. QAF imaging was repeated three times, with brief intervals and new adjustments of the camera in between. The image series with the best image quality (e.g. evenly distribution of the light, focus on the fovea, image centered at the fovea) was taken for further analysis.

### Image Analysis

The Pentacam Nucleus Staging (PNS) Grading score was extracted from the Scheimpflug device´s software for analysis (PNS and 3D cataract analysis package, Pentacam).^17^^,^^18^ PNS provides information on the mean lens density value, standard deviation, and maximum nucleus lens density and subdivides it on a scale from 0 to 5 a.u. (exact formula not published by the manufacturer). The grey values of the AC-OCT images were normalized to values between 0 and 1 using ImageJ as previously published (white = 1 and black = 0).^19^ In the next step, the relative reflectivity of the lens compared to the cornea was calculated. The LQAF images were imported into ImageJ as a stack of 64 bitmap images, and the LQAF was calculated according to the study protocol of Charng and colleagues using the provided formula.^5^ Briefly, the highest LQAF value from all slabs (out of 64) of the z-stack was measured in a 60 × 60-pixel region in the center of the image and divided by the autofluorescence measurement from a separate 200 × 18-pixel region of the internal reference. Finally, the QAF image of the retina was analyzed using custom-written Fiji plugins, as recently described.^16^ Briefly, the OCT image was registered to the QAF images using vessel bifurcations. This allowed the alignment of pre-fabricated QAF analysis grids to precisely measure QAF at the same location in each participant.^20^ We chose the QAF8 ring defined by Greenberg and colleagues as it has been widely accepted for QAF intensity analysis.^4^^,^^7^^,^^21^ Finally, the mean value of the QAF8 ring, located at 6 degrees to 8 degrees eccentricity, was determined and extracted for statistical analysis.

### Statistical Analysis

Among the subset of participants undergoing cataract surgery, least absolute shrinkage and selection operator (LASSO) regression (glmnet package in R) and backward selection were implemented to determine which variable – age, LQAF, PNS, or AC-OCT – or a combination of variables best predicts the QAF value after lens extraction.^22^ Predictors were normalized, specifically mean-centered, and scaled by their standard deviation, to ensure that each variable contributed equally to the regression analysis, avoiding bias due to differing scales. The selection of the regularization parameter in the leave-one-out cross-validation was meticulously conducted with 100 different parameters considered, spanning a range from a lambda value at which all coefficients shrink to zero (indicating no overfitting), to a lambda value that equates the model's fit to that of a simple linear regression (providing a balance between model complexity and prediction accuracy). Through this process, LASSO regression inherently performs feature selection by penalizing the absolute size of the coefficients, which serves a similar purpose to traditional backward selection but is achieved automatically within the LASSO framework. Additionally, the mean absolute error (MAE) of the age-based QAF value pre-surgical with the QAF value post-surgical (without correction) were calculated as a reference for the accuracy of the current approach. Finally, a spline model was applied to quantify the impact of LQAF and PNS on QAF in a large patient cohort (mgcv package in R). The model consisted of one spline showing the increase in QAF as a function of age combined with two interaction splines that include a smoother term of age according to the LQAF/PNS value. The *P* values were also calculated by the spline model to determine the statistical significance of the observed relationships (*P* < 0.05 considered to be statistically significant).

## Results

### Baseline Characteristics

A total of 130 eyes of 130 participants, 49% of them women, were included in this study at baseline (mean age 63 ± 17 years, range = 21–87 years; Table 1). Twenty-three participants had to be excluded from the analysis because of poor QAF image quality, which was mainly due to severe cataracts. Thirty participants who had routinely scheduled cataract surgery received duplicate QAF imaging before and 2 weeks after surgery. The imaging protocol was performed as described, except for the AC-OCT imaging, which was only performed in a subgroup of 90 subjects due to the availability of the imaging device.

**Table 1.** Study Population

Characteristic	Value
Participants, *n*	130
Age, years, mean, SD, [range]	63 ± 17 [21–87]
Gender	
Female	49%
Male	51%
Laterality eye	
Right	55%
Left	45%
PNS mean, SD	1.53 ± 1.18 a.u.
0	17%
1	40%
2	26%
3	11%
4	2%
5	4%
Reflectivity ANTERION, mean, SD, [range]	4.26 ± 1.03 [2.11–6.81] a.u.
LQAF 1 mean, SD, [range]	15.90 ± 6.65 [2.59–28.88] a.u.

LQAF, lenticular quantitative autofluorescence; PNS, Pentacam nucleus score; SD, standard deviation; y, year.

### Accuracy of Current Age-Based Correction Without Lens Scores

**Figure 1.**
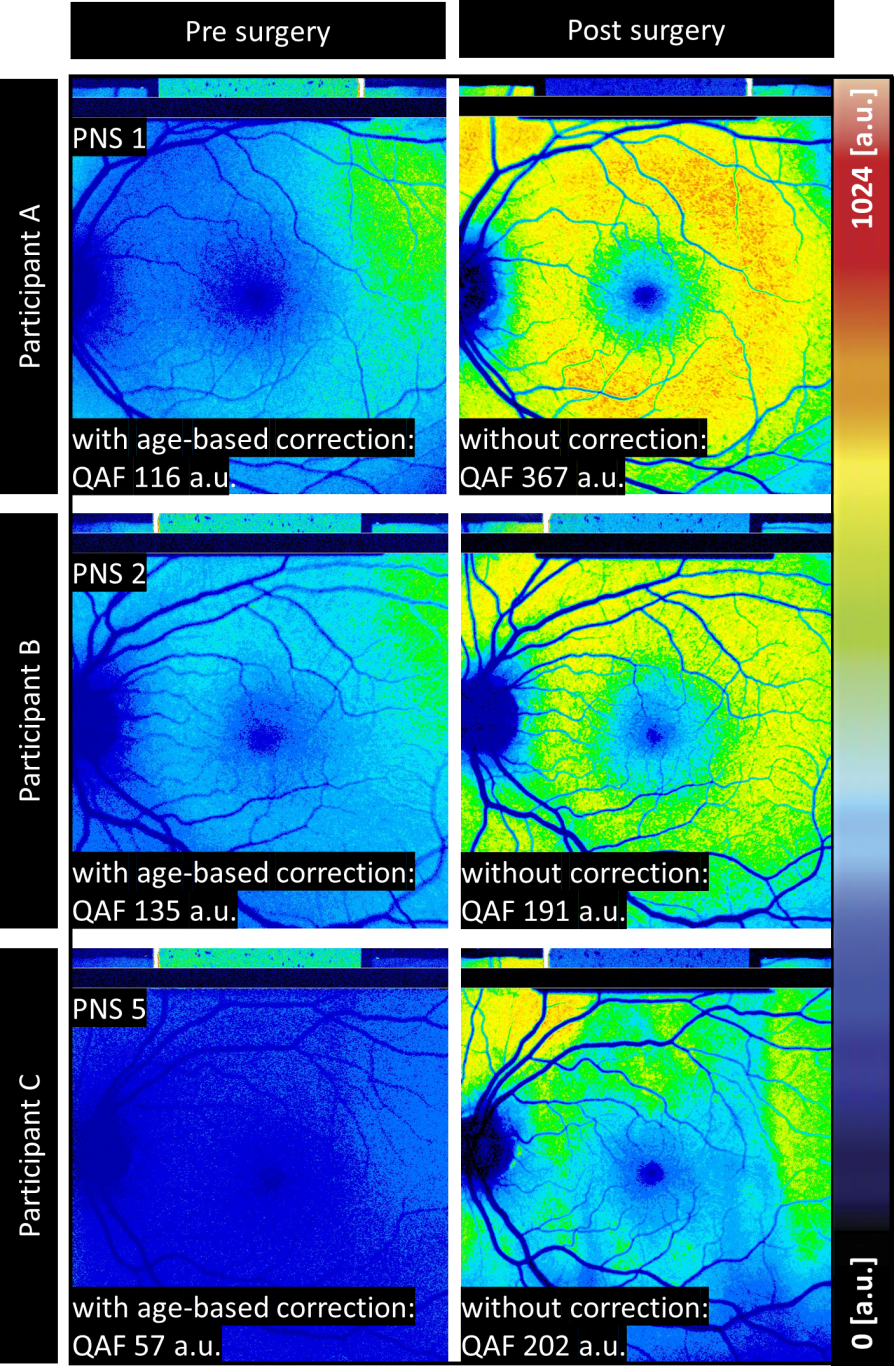
**QAF images pre and post cataract surgery of three different patients with varying PNS scores**. Three different participants were compared (**A**) a 56-year-old woman, (**B**) a 45-year-old man, and (**C**) a 57-year-old woman. The actual measured QAF values without age correction of the lens are reported post-surgically and compared to the age-based estimate of the QAF value under the impact of the opacified lens pre-surgically. In the images recorded post-surgically, age correction of the lens is not necessary, as the inserted artificial lens is free of any opacification. The mean absolute error of the currently used age-based correction was 96.1 ± 93.5 MAE ± SD (QAF a.u.).

To determine the accuracy of the currently deployed age-based correction, we compared QAF values in subjects before and after lens extraction. The formula only applies a correction factor for participants with a natural lens and, therefore, if the formula is accurate, the difference between the two measurements should be miniscule. The accuracy of the currently used age-based correction was 96.1 (QAF a.u.) MAE with an SD of 93.5 (QAF a.u.) (Fig. 1).^4^ Cataract-operated participants had a mean value of lens score of 2 for PNS, AC-OCT score of 4.89 (a.u.), and an LQAF score of 18.8 (a.u.).

### Lenticular Opacification and Autofluorescence

**Figure 2.**
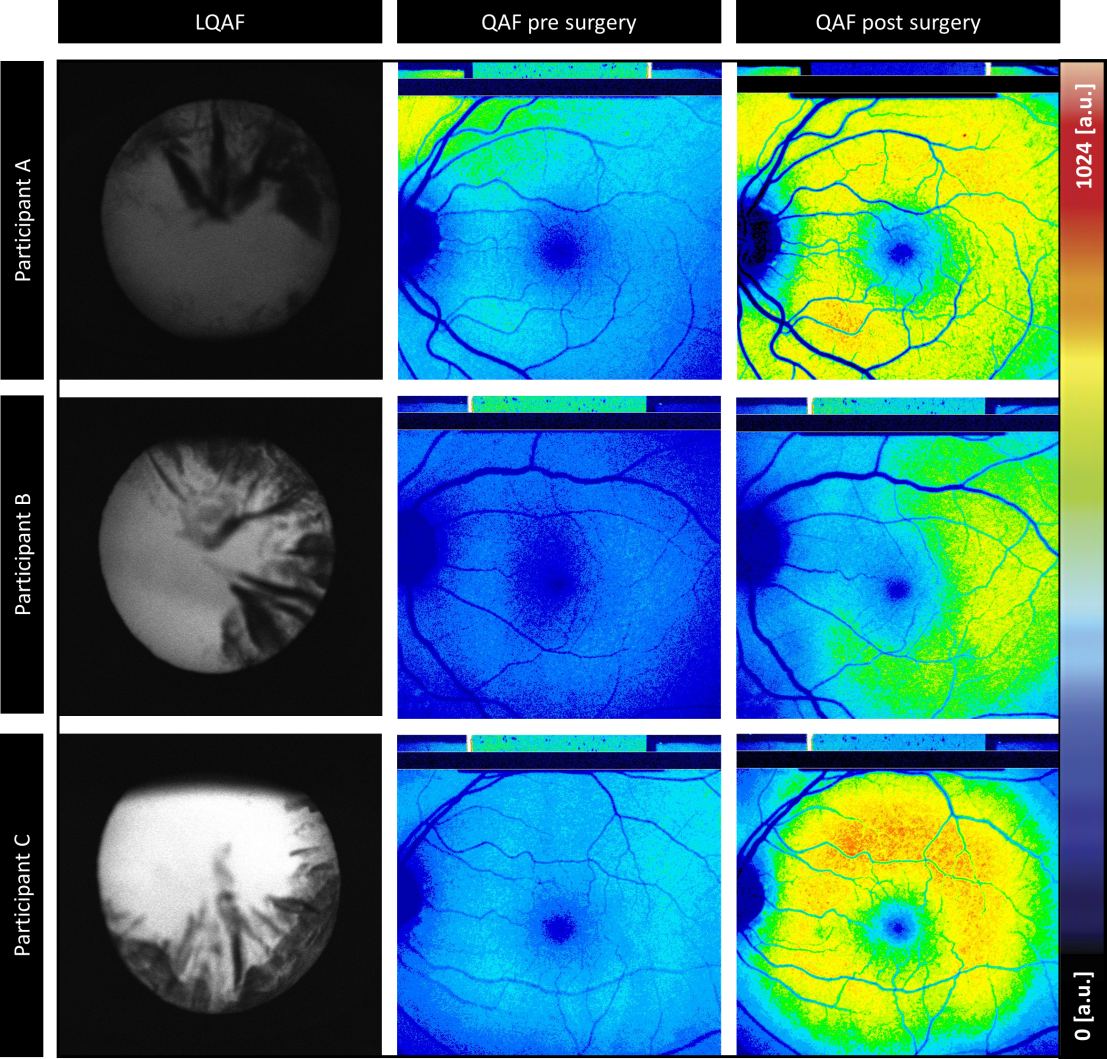
**Different lens opacity patterns in lenticular quantitative autofluorescence (LQAF) imaging**. In three different patients and their associated quantitative autofluorescence (QAF) images pre- and post-cataract surgery (**A**) a 48-year-old man, (**B**) a 75-year-old woman, and (**C**) a 77-year-old woman. Despite the different lens opacity patterns, the QAF image quality in all three images is uniformly attenuated across the entire image.

The mean values for the lens scores, LQAF, PNS, and Reflectivity AC-OCT were mean ± SD (95% quartiles) = 15.90 ± 6.65 (95% quartiles = 2.59–28.88), 1.53 ± 1.18 (95% quartiles = 1–5), and 4.26 ± 1.03 (95% quartiles = 2.11–6.81) a.u., respectively. Different lens opacity patterns could be detected in the LQAF measurements. However, no regional aberrations could be found in the corresponding QAF images, the image quality was uniformly degraded across the entire image (Fig. 2).

### Most Significant Lens Scores for QAF Prediction

**Figure 3.**
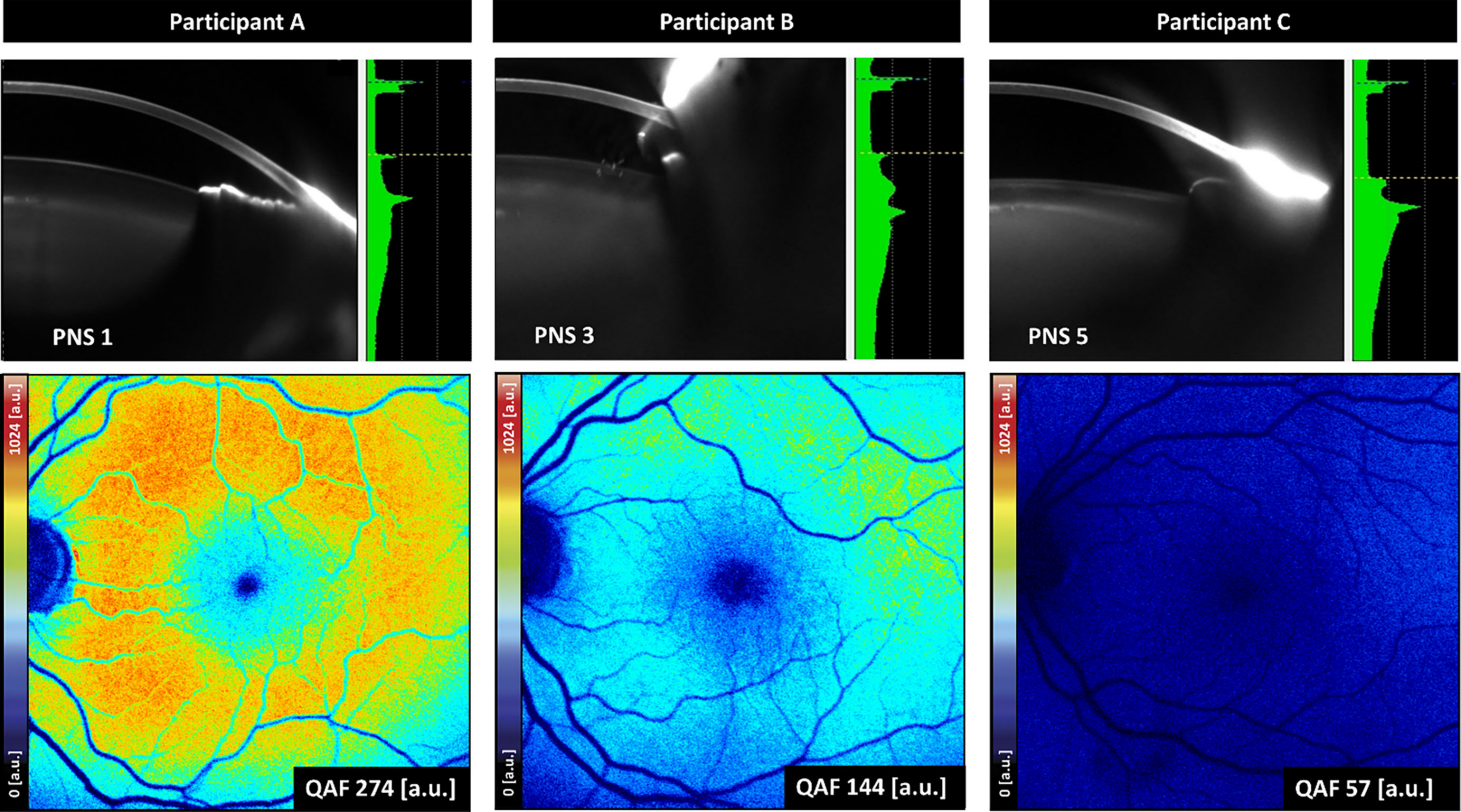
**Comparison of the relationship of lenticular** **opalization** **and QAF values.** Images of three different participants are shown, each with the Pentacam nucleus score (PNS) image on top and the corresponding quantitative autofluorescence (QAF) image below (**A**) a 86-year-old man, (**B**) a 77-year-old woman, and (**C**) a 57-year-old woman. The PNS images demonstrate various lens opacities. Color-coded QAF images show different QAF intensities depending on the lens opacity (*black/blue* = low QAF values and *red/white* = high QAF values). The QAF image on the *left* with a corresponding lens score of PNS 1 (= low lens opacity) show the highest QAF intensity (274 a.u.). In comparison the QAF image on the *right* with a corresponding lens score of PNS 5 (= high lens opacity) shows the lowest QAF intensity (57 a.u.). Increasing PNS values demonstrate a decrease in QAF values.

LASSO regression analysis revealed age and LQAF measurements as the most relevant variables to predict QAF differences pre- and post-cataract surgery, whereas first AC-OCT measurements and then PNS were eliminated by backward selection (cross-validated R2 = 0.49). Both PNS and LQAF were associated with a decrease in QAF values (Fig. 3).

### QAF Prediction Accuracy Using Lens Scores

The prediction accuracy of the spline mixed model was MAE 32.2 (QAF a.u.). The addition of lens scores for the QAF prediction could markedly reduce the measured prediction error compared to the age-based method. Both smooth terms, LQAF1 and PNS, were significant for the spline model, with *P* < 0.01 and *P* < 0.01, respectively (Table 2, Supplementary Fig. S1). This suggests that age has a significant interaction with both LQAF1 and PNS on QAF measurements. A linear regression model of LQAF and QAF for comparison only yielded an R2 of 0.15.

**Table 2.** Spline Model Results

Measure	Intercept	S (Age)	S (Age): LQAF	S (Age): PNS
Estimate	148.64	–	–	–
Std. error	12.63	–	–	–
*T* value	11,77	–	–	–
*P* value	<0.001	0.59	<0.01	<0.001
Edf	–	1.5	2	2
Ref. df	–	1.9	2	2
F-statistic	–	0.56	5.35	5.16

This table presents the results of a spline model analysis for quantitative autofluorescence (QAF) prediction using lens scores. The first section of the table displays the measure of each coefficient, its estimated value, standard error, *t* value, *P* value, estimated degrees of freedom (edf), reference degrees of freedom (Ref. df), and F-statistic, if applicable. The variable “Intercept” represents the estimated starting point or the baseline value when all other independent variables are zero. The spline smoothed variables represent the lens scores quantitative autofluorescence (LQAF) and Pentacam nucleus score (PNS) with their respective age interaction. The overall fit of the model was R^2^ 0.34 (36.3% Deviance explained). The model could predict QAF with a mean absolute error of 32.2 and a standard deviation of 23.4.

## Discussion

Our results show that lens scores, LQAF1, PNS, and reflectivity AC-OCT can be used to quantitatively describe the lens opacity and predict its impact on retinal QAF measurements. An increase in both PNS and LQAF lead to decreased QAF values, indicating that lenticular opacification, rather than intrinsic autofluorescence, is the driving factor for the impact of the lens on QAF imaging. Compared to the current practice of age-based correction, individualized lens-based correction could increase the prediction accuracy of QAF measurements.^4^

In concordance with previous work, we demonstrate that lenticular aging varies greatly between individuals.^5^^,^^6^ Several aspects are described that can cause interindividual differences in the onset of age-related cataracts, such as genetic and environmental factors like smoking, diabetes, uveitis, ultra-violet light and sun exposure, steroid usage, trauma, and intraocular surgery.^23^ In our cohort of participants without a history of ocular disease, smoking, genetic factors, and sun exposure are most likely the main factors. The main cause of lens opacity is considered to be the aggregation of lens crystalline proteins.^24^ There are three main types of crystalline proteins: a, ß, and y-crystalline protein, with native-sized a-crystalline proteins acting as chaperone-like molecules, thereby having a protective effect on the lens cell.^24^ This effect is lost in the aging lens, where abnormal cross-linked a-crystalline proteins form, most likely due to oxidative stress, resulting in the development of nuclear and cortical cataracts.^24^^,^^25^ The aggregates lead to a discontinuity in the refractivity of the lens and cause light scattering, an increased spectral absorption, especially for short-wavelength blue light, and a loss of light transmission to the retina.^3^^,^^24^ Apart from opacification, fluorophores (e.g. 3-OH-l-kynurenine-O-β-glucoside) of the lens also accumulate with age that could, in theory, have an additive effect on retinal autofluorescence images.^26^^,^^27^

Incorporating color photography into lens assessment could significantly enhance the accuracy of lens score derivation, which currently relies on black and white images.^18^ This method may neglect critical information about the lens's color, particularly in cases of advanced cataract brunescence. The brunescence, characterized by the yellowing or browning of the lens, can markedly affect light transmission, especially in the blue spectrum.^28^ This might also impact QAF measurements. Color images would enable direct observation and quantification of this color change (yellowing or browning with age), providing a more comprehensive understanding of the lens's condition and potentially improving the precision of QAF predictions in elderly persons with cataract. Adding this dimension to lens assessment could refine correction formulas used in QAF imaging, ensuring a more accurate evaluation of retinal health.

In our study, as well as in a recent publication by Reiter and colleagues, we could show that lenticular opacification supersedes the intrinsic autofluorescence signal of the lens.^1^ Nonetheless, LQAF proved to be the most predictive lens score in the LASSO regression model, and an increase in LQAF was associated with reduced retinal autofluorescence. However, whether LQAF is a sole surrogate marker for lenticular opacification or intrinsic lenticular AF plays a role in QAF imaging remains to be determined.

When selecting the best imaging modality to quantify the impact of the aging lens on QAF images, we must consider the different possible cataract types. Clinically, cataracts can be divided into three categories with anatomic differences: nuclear, cortical, and posterior subcapsular, whereas nuclear cataracts are most common. Reiter et al. recently described a correlation between the severity of lens opacity presurgically and the increase of the QAF postsurgically, showing that the lens opacity contributes more to light blocking than adding to the retinal autofluorescence signal.^1^ The results were only significant for cortical cataracts but showed a trend toward QAF increase after surgery also for nuclear cataracts.^1^ Reflecting on the imaging modalities used in this study, there are differences in the accuracy depending on the cataract type. For the LQAF measurements, we used a 60 × 60 pixels square in a central slab of the LQAF z-stack.^5^ Therefore, opacities that are more peripheral, which is typical for the cortical cataract, may be missed. In the AC-OCT image, we measured the lens density in a central section axial to the cornea, leading to only a small part of the lens that was included in the calculation. With this method, both nuclear cataracts and posterior subcapsular opacities could be detected, but to a lesser degree, cortical cataracts. Only the Pentacam nucleus grading score (PNS), which uses a three-dimensional image of the whole lens, may be able to detect all three cataract types.^29^ The QAF imaging in our study was performed using a cSLO device. One significant advantage of using cSLO for QAF imaging is the pinhole effect, which we believe minimizes the impact of lens inhomogeneity on the correction. This effect ensures that only light from the focal plane reaches the detector, reducing the influence of scattered light and potentially providing more accurate autofluorescence measurements.

Considering that QAF imaging uses confocal scanning lasers (placement of a pinhole at a conjugate plane before the detector), we hypothesize that peripheral parts of the lens do not contribute to the QAF image. This theory is also supported by Figure 2, where different patterns of lenticular opacification seemed not to transfer on the QAF image that was uniformly opaque.

To better achieve prediction performance and identify the most important areas of lenticular opacification, alternate approaches could have been beneficial. For instance, a deep learning approach could have been applied, which would leverage the raw LQAF, Pentacam, and AC-OCT images directly. Instead of reducing these complex images to simple numerical lens scores, deep learning methods could explore the full potential of these imaging techniques by analyzing them in their raw, high-dimensional form. Some methods that were already investigated use slit lamp photographs and grade the cataract severity automatically by using a neural network that uses predefined landmarks on the visual axis.^30^^,^^31^ An extension of this system was trained to analyze the entire lens structure based on features such as intensity, color, and entropy.^32^ In comparison with expert graders, these methods have already achieved reliable results.^10^ The use of a deep learning approach would, however, have come at the cost of applicability, as the current lens scores can be easily reproduced using open-access software.

To better evaluate the predictive power of our lens correction factor, we need to relate it to the consistency of QAF imaging. The QAF retest variability between visits ranges between an intraclass correlation coefficient (ICC) of 0.76 to 0.93.^33^^,^^34^ The QAF prediction model is therefore limited in the accuracy that is possible to achieve. Currently, we could achieve a deviance explained of 36.3% in our prediction model. One impediment to prediction accuracy could be the inclusion of cataract participants who were scheduled for surgery. We presume that the predictive power might have been lower in a cohort of only mild lens opacities. Similarly, the prediction accuracy of the current age-based correction factor was only evaluated in participants with severe cataracts. It is possible that the age-based correction factor is more accurate across a broader patient cohort.

To easily apply our lens scores for QAF value calculation, the integration of our lens correction into the imaging software would be desirable. In particular, the Pentacam nucleus score promises easy, time-efficient use, as the image acquisition takes only a few seconds, and the degree of lens opacity is immediately displayed internally without the need to calculate it separately.^15^ By contrast, both the LQAF and anterior chamber OCT score currently require post-processing which is more time-consuming. A possible solution for this could be customized plugins to allow automated calculation.^35^ Another promising approach would be to use deep learning methods to estimate the lens opacity based on the image quality of the retinal image. In this way, only one QAF image of the retina would be needed to determine the lens score.^36^^,^^37^

Limitations of this study include the relatively small number of controls (*n* = 30) with QAF data with and without the natural lens. Further, as mentioned before, we could only include participants with severe cataract into this cohort. Strengths of this study are the extensive multimodal imaging for the lens as well as the retina deployed, the large and diverse sample of patients that encompass individuals of all age groups, and cataract severity, as well as the exclusive use of the same type of clear intraocular lens for all patients (to exclude variations expected by the implant itself).

In conclusion, the introduction of lens scores, LQAF, PNS, and reflectivity in AC-OCT could lead to more accurate quantification of lenticular impact on retinal QAF imaging. LQAF and PNS were the most important variables. By validating the lens scores against actual measured QAF values without the impact of the natural lens, we could show that the predictive power for our models exceeds that of the previous age-based lens correction factor. The application of lens scores in the clinic could lead to an improvement of the accuracy of QAF image interpretation and might also enable inclusion of aged patients with phakic lens in future QAF studies.

## Supplementary Material

Supplement 1
