# Supplementary material for: Personalized Lens Correction Improves Quantitative Fundus Autofluorescence Analysis
Source: Invest Ophthalmol Vis Sci. 2024 Mar 11;65(3):13. doi: 10.1167/iovs.65.3.13 (PMC10929741; doi:10.1167/iovs.65.3.13)
Supplement: Supplement 1 [file iovs-65-3-13_s001.pdf]

Supplementary figure 1

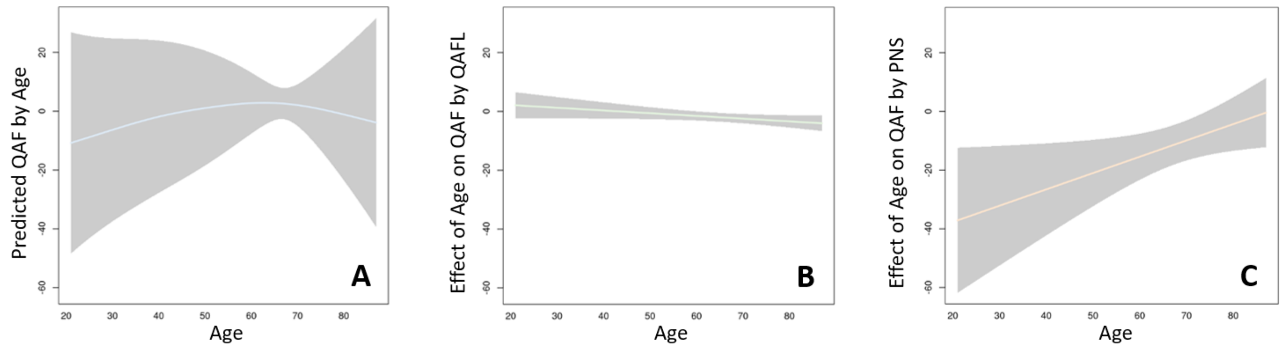

Supplementary figure 1: Spline graphs of the effect and interaction of age and lens scores on quantitative autofluorescence of the fundus.

Supplementary figure 1 shows the splines applied to the three age-related input features. Graph A captures the effect of age on QAF values. The y-axis displays the age-spline dependent on age in the x-axis. The large standard error estimates (and subsequent wide confidence bands) suggest that no clear relationship can be inferred, and significant difference from zero cannot be assumed for this term (p=0.75). Graph B displays the relationship between age and QAF depending on QAFL. The graph is linear with estimated degrees of freedom (edf) of 2. The p-value of 0.03 suggests a relationship between age and QAF dependent on QAFL specifically that in patients with high lenticular autofluorescence QAF slightly declines with age. The narrow 95% confidence interval (using standard errors and assuming a normal distribution for the estimate) showcases the small standard error estimate. Similarly, Graph C shows how across the entire age range, a higher PNS score is associated with lower QAF, but the strength of this relationship decays with age. The p-value < 0.01 suggests that PNS modifies the age-QAF relationship significantly.

In summary, while age on its own does not seem to have a significant relationship with QAF, the interactions suggest that the relationship between age and QAF does change depending on the values of QAFL and PNS.
